# Predictors of Length of Stay and Cost of Hospitalization of Neonatal Abstinence Syndrome in the United States

**DOI:** 10.7759/cureus.16248

**Published:** 2021-07-07

**Authors:** Parth Bhatt, Jacob Umscheid, Narendrasinh Parmar, Rhythm Vasudeva, Kripa G Patel, Akosua Ameley, Keyur Donda, Brian Policano, Fredrick Dapaah-Siakwan

**Affiliations:** 1 Pediatrics, United Hospital Center, Bridgeport, USA; 2 Pediatrics, University of Kansas School of Medicine - Wichita, Wichita, USA; 3 Pediatrics, Brookdale University Hospital Medical Center, Brooklyn, USA; 4 Internal Medicine/Pediatrics, University of Kansas, Wichita, USA; 5 Pediatrics, Smt. Nathiba Hargovandas Lakhmichand Municipal Medical College, Ahmedabad, IND; 6 Pediatrics, Greater Accra Regional Hospital, Accra, GHA; 7 Pediatrics/Neonatology, University of South Florida, Tampa, USA; 8 Neonatology, Valley Children’s Healthcare, Madera, USA

**Keywords:** neonatal abstinence syndrome, length of stay, cost of hospitalization, predictor, nas

## Abstract

Background

The incidence rate and economic burden of neonatal abstinence syndrome (NAS) are increasing in the United States (US). We explored the link between the length of stay (LOS) and hospitalization cost for neonatal abstinence syndrome in 2018.

Methods

This was a cross-sectional analysis of the 2018 national inpatient sample database. Newborn hospitalizations with neonatal abstinence syndrome and their accompanying comorbid conditions were identified using the International Classification of Diseases, 10th Edition diagnostic codes. Logistic regression was used to determine the impact of length of stay and the co-morbidities on inflation-adjusted hospital costs.

Results

The incidence of neonatal abstinence syndrome was 7.1 per 1000 births (95% CI 6.8-7.3) in 2018. The majority had Medicaid (84.1%), with a neonatal abstinence syndrome incidence of 13.2 (95% CI: 12.8-13.6). In adjusted analysis, every one-day increase in length of stay increased the hospital cost by $1,685 (95% CI: 1,639-1,731). Neonatal abstinence syndrome hospitalizations with Medicaid had a longer length of stay by 1.8 days (95% CI: 0.5-3.1). Co-morbidities further increased the length of stay: seizures: 13.8 days; sepsis: 4.1 days; respiratory complications: 4.4 days; and feeding problems: 5.8 days. Those at urban teaching hospitals had a longer length of stay by 7.3 days (95% CI: 5.8-8.8). Co-morbidities increased hospital cost as follows: seizures: $71,380; sepsis: $12,837; respiratory complications: $8,268; feeding problems: $7,737. The cost of hospitalization at large bed-size hospitals and urban teaching was higher by $5,243 and $12,005, respectively.

Conclusion

The incidence rate of neonatal abstinence syndrome remained high and was resource-intensive in 2018. Co-morbid conditions and hospitalization at urban teaching hospitals were major contributors to increased length of stay and hospital costs.

## Introduction

Neonatal abstinence syndrome (NAS) is a clinical diagnosis and a consequence of the abrupt discontinuation of chronic fetal exposure to substances that were used or abused by mothers during pregnancy [1]. Between 2010 and 2017, the rate of NAS increased nationally. Newborns with NAS had longer lengths of stay (LOS) by nine days and $14,600 in additional costs in 2017 [2]. While previous studies have examined resource use in NAS, the direct link between LOS and hospital costs has not been studied [3,4]. Further, the predictors of LOS and increased hospital costs have not been extensively studied. To date, there are no national studies describing predictors of length of stay and cost of hospitalization among NAS hospitalizations. To address this gap, we used a nationally representative dataset to study (a) incidence and distribution of NAS within the United States, (b) cost of hospitalization as a function of LOS, and (c) predictors of LOS and cost of hospitalization among NAS hospitalizations.

## Materials and methods

We conducted a retrospective cross-sectional cohort analysis of the 2018 Healthcare Cost and Utilization Project’s National Inpatient Sample (NIS) database. Newborn hospitalizations with NAS were identified with International Classification of Diseases, Tenth Revision, Clinical Modification (ICD-10-CM) codes 'P96.1' and 'P04.14'. The exposure variable was NAS, and the outcomes of interest were the incidence rate of NAS according to demographics and the resource utilization as measured by LOS and hospital costs. Hospital charges were converted to costs using Healthcare Cost and Utilization Project hospital-specific cost-to-charge ratios. Costs reflect the actual expenses incurred during the inpatient stay [5,6]. Co-morbid conditions such as preterm birth, respiratory complications, feeding complications, sepsis, seizure, low birth weight were identified using relevant ICD-10-CM codes [4,7]. The NIS has a complex survey design. To calculate predictors of LOS and cost of hospitalization, hierarchical regression models were used. Two-level hierarchical models [smaller unit factors (patients) nested within larger unit factors (hospital)] were created, with the unique hospital identification number incorporated as a random effect within the model. Hospital identification was incorporated as a random effect in the model to account for the impact of hospital clustering to account for possibly similar outcomes in patients being treated at the same hospital due to certain processes of care received [8]. SAS 9.4 (SAS Institute, Cary, NC) was used for all analyses. This study included publicly available deidentified data and thus was exempt from review by the institutional review board.

## Results

In 2018, the overall incidence of NAS was 7.1 per 1000 births (95% CI 6.8-7.3). Majority of infants with NAS had Medicaid (84.1%) (95% CI: 82.8-85.3) with incidence of 13.2 (95% CI: 12.8-13.6) (Table 1).

**Table 1:** Characteristics and rates of infants receiving a diagnosis of neonatal abstinence syndrome vs. all other US hospital births, 2018 LOS: length of stay; NAS: neonatal abstinence syndrome; CI: confidence interval

	NAS rate per 1000 births	NAS n = %, (95% CI)	All other newborn hospitalizations n = %, (95% CI)	P-value
Overall	7.1 (6.8-7.3)			
Sex				0.02
Male	7.4 (7.1-7.6)	52.9 (51.7-54.2)	51.0 (50.9-51.1)	
Female	6.8 (6.6-7.0)	47.0 (45.7-48.3)	49.0 (48.8-49.1)	
Race/ Ethnicity				< .0001>
White	10.7 (10.3-11.0)	69.8 (67.7-71.9)	46.2 (44.9-47.4)	
Black	4.1 (3.7-4.4)	7.7 (6.7-8.7)	13.4 (12.7-14.1)	
Hispanic	2.8 (2.5-3.0)	7.2 (6.1-8.2)	18.5 (17.4-19.5)	
Native American/ Alaska native	0.5 (0.3-0.8)	0.4 (0.2-0.6)	5.4 (4.9-5.9)	
Other	5.2 (4.5-5.8)	5.4 (4.3-6.5)	7.4 (6.8-7.9)	
Household income, quartile				< .0001>
1st	9.5 (9.0-9.5)	35.9 (33.6-38.2)	26.8 (25.7-27.9)	
2nd	8.2 (7.9-8.6)	30.0 (28.3-31.7)	25.7 (24.9-26.5)	
3rd	6.2 (5.9-6.5)	21.5 (20.0-23.1)	24.5 (23.8-25.2)	
4th	3.7 (3.5-3.8)	11.4 (10.2-12.6)	22.1 (20.8-23.4)	
Payment				< .0001>
Medicaid	13.2 (12.8-13.6)	84.1 (82.8-85.3)	44.7 (43.6-45.9)	
Private	1.5 (1.4-1.6)	10.0 (9.0-11.0)	47.1 (45.9-48.3)	
Uninsured	6.2 (5.6-6.6)	4.4 (3.7-5.1)	5.1 (4.7-5.5)	
Other	3.3 (2.7-3.8)	1.3 (1.0-1.7)	2.9 (2.7-3.1)	
Hospital bed-size				0.05
Small	6.8 (6.5-7.1)	18.7 (16.7-20.6)	19.5 (18.3-20.7)	
Medium	6.4 (6.0-6.7)	27.6 (25.1-30.2)	30.6 (29.2-31.9)	
Large	7.6 (7.3-7.9)	53.7 (50.8-56.6)	49.9 (48.4-51.5)	
Urban/ Rural				< .0001>
Rural	10.5 (9.1-11.7)	13.2 (11.1-15.2)	9.0 (8.5-9.4)	
Urban non-Teaching	6.2 (5.8-6.5)	16.8 (14.9-18.6)	19.4 (18.4-20.5)	
Urban teaching	7.0 (6.7-7.2)	70.1 (67.5-72.6)	71.6 (70.4-72.7)	
Delivery hospital region				< .0001>
Northeast	9.4 (8.9-9.9)	21.0 (18.6-23.5)	15.7 (14.6-16.9)	
Midwest	6.6 (6.1-7.0)	19.7 (17.5-21.9)	21.3 (20.1-22.4)	
South	7.6 (7.2-7.9)	42.2 (39.3-45.2)	39.5 (37.9-41.0)	
West	5.1 (4.8-5.4)	17.1 (15.2-18.9)	23.5 (22.3-24.8)	

Infants with NAS had a mean, standard deviation (SD) LOS of 14.5 days (± 0.3), costs of $17,590 (± 630) per hospitalization, and aggregate cost for NAS amounted to $449.1 million in 2018. In adjusted analysis, every one-day increase in length of stay increased the cost of hospitalization by $1,685 (95% CI: 1,639-1,731) (Table 2).

**Table 2:** Multivariate predictor for the cost of hospitalization among newborn with neonatal abstinence syndrome

Variable	Beta co-efficient, 95% Confidence Interval	P-value
Length of stay; one-day increase ($, Unadjusted)	1713 (1668 - 1758)	<0.0001
Length of stay; one-day increase ($, Adjusted)	1685 (1639 - 1731)	<0.0001
Hierarchical regression adjusted for gender, race, gestational age, low birth weight, respiratory complications, feeding complication, sepsis, seizure, hospital region, insurance type, hospital location, and teaching status, hospital bed-size		

On multivariable regression analysis, NAS hospitalizations with Medicaid had longer LOS by 1.8 days (95% CI: 0.5-3.1). Co-morbidities further increased the LOS among NAS hospitalizations: seizures: 13.8 days (95% CI: 9.1-18.4), sepsis: 4.1 days (95% CI: 1.7-6.5), respiratory complications: 4.4 days (95% CI: 3.5-5.3); and feeding problems: 5.8 days (95% CI: 4.8-6.7). NAS hospitalizations at large bed-size had longer LOS by 3.0 days (95% CI: 1.7-4.3) compared to hospitalizations at small bed-size. Urban teaching NAS hospitalizations had longer LOS by 7.3 days (95% CI: 5.8-8.8) compared to rural hospitalizations. No significant differences in cost were observed between those with and without Medicaid. Multivariable analysis for cost of hospitalization showed similar increases in cost for co-morbidities: seizures: $71,380 (95% CI: $60,792-$81,968); sepsis: $12,837 (95% CI: $7,294-$18,380); respiratory complications: $8,268 (95% CI: $6,115-$10,421); feeding problems: $7,737 (95% CI: $5,471-$10,002). Cost of hospitalization at large bed-size hospitals and urban teaching was higher by $5,243 (95% CI: $1,587-$8,898) and $12,005 (95% CI: $7,915-$16,095), respectively (Table 3).

**Table 3:** Predictor of length of stay and cost of hospitalization among newborns with neonatal abstinence syndrome, 2018

Variables	Length of Stay (days)		Cost of hospitalization (US$)	
	Beta-coefficient (95% CI)	P-value	Beta-coefficient (95% CI)	P-value
Intercept	2.1 (-0.2 – 4.4)	0.08	2718 (-3361 – 8798)	0.4
Gender				
Male			Reference	
Female	- 0.1 (-0.9 – 0.6)	0.7	405 (-1358 – 2167)	0.7
Race/ Ethnicity				
White	Reference	Reference	Reference	Reference
Black	-0.5 (-1.9 – 0.9)	0.5	-172 (-3421 – 3077)	0.9
Hispanic	1.1 (-0.4 – 2.6)	0.2	826 (-2711 – 4363)	0.6
Asian/ Pacific Islander	1.2 (-4.8 – 7.2)	0.7	14346 (181 – 28511)	0.05
Native American/ Alaska native	0.8 (-2.3 – 3.9)	0.6	141 (-7325 – 7607)	0.9
Preterm	0.9 (-0.3 – 2.1)	0.1	3031 (289 – 5774)	0.03
Low birth weight	2.2 (0.9 - 3.4)	0.0007	3528 (675 – 6381)	0.02
Respiratory complication	4.4 (3.5 – 5.3)	< .0001>	8268 (6115 – 10421)	< .0001>
Feeding complication	5.8 (4.8 – 6.7)	< .0001>	7737 (5471 – 10002)	< .0001>
Sepsis	4.1 (1.7 – 6.5)	0.001	12837 (7294 – 18380)	< .0001>
Seizure	13.8 (9.1 – 18.4)	< .0001>	71380 (60792 – 81968)	< .0001>
Hospital region				
Northeast	Reference	Reference	Reference	Reference
Midwest	-0.9 (-2.5 – 0.7)	0.3	-2815 ( -7252 – 1622)	0.2
South	0.5 (-0.9 – 1.9)	0.4	-4856 (-8749 – -962)	0.01
West	0.0 ( -1.7 – 1.7)	0.9	7098 (2488 – 11707)	0.003
Insurance type				
Private	Reference	Reference	Reference	Reference
Medicaid	1.8 (0.5 – 3.1)	0.007	-1924 (-5013 – 1165)	0.2
Self-pay/ Others	-1.5 (-3.6 – 0.7)	0.2	-6656 (-11666 – -1645)	0.01
Hospital bed-size				
Small	Reference	Reference	Reference	Reference
Medium	1.7 (0.3 – 3.2)	0.02	1401 (-2494 – 5296)	0.5
Large	3.0 (1.7 – 4.3)	<0.0001	5243 (1587 – 8898)	0.005
Urban/ Rural				
Rural	Reference	Reference	Reference	Reference
Urban non-teaching	4.7 (2.9 – 6.5)	< .0001>	5781 (1042 – 10520)	0.02
Urban teaching	7.3 (5.8 – 8.8)	< .0001>	12005 (7915 – 16095)	< .0001>

## Discussion

Using a nationally representative database, we found that in 2018, the NAS incidence remained high and continued to be resource-intensive. Co-morbid conditions, hospitalization at urban teaching hospitals, and large bed size hospitals are major contributors to higher LOS and costs. To our knowledge, this is the first study that showed that each day increase in LOS increased hospital cost by $1,685.

Our finding of the NAS incidence of 7.1 per 1000 live births in 2018 is consistent with a previous report from the HCUP that showed the incidence to 7.0 and 7.3 in 2016 and 2017, respectively [5]. Taken together, this suggests that the incidence of NAS may be plateauing but further surveillance is required to ascertain this.

Our estimates of mean LOS (14.5 days) and cost of hospitalization ($17,590) were lower compared to previous estimates [4,9]. This difference could be due to differences in population derivation or different time periods. Nevertheless, trends in LOS and cost need to be studied further.

NAS hospitalizations with co-morbid conditions such as seizure, sepsis, respiratory complications, feeding complications, and low birth weight were found to have higher LOS and hospitalization costs. This is similar to previous studies and is explanatory [3,7]. We found higher LOS and cost of hospitalization among NAS hospitalizations at urban teaching and large bed size. One of the reasons for this finding may be secondary to care within the neonatal intensive care unit (NICU) at these hospitals. Large variation exists in care for newborns with NAS. Moreover, Grossman et al. showed that infants with NAS treated on the general inpatient floor had shorter LOS compared to those who were treated in NICU [10]. We were not able to assess this as the NIS does not allow identification of NICU admissions from general inpatient floor admissions. Another reason for this finding may be due to severe cases being managed at these centers and thus longer LOS and higher costs. Further research is warranted to explore this finding. Lastly, we report the cost of NAS hospitalization as a function of LOS. A reduction in LOS will directly reduce hospital costs and this should give impetus to clinicians, administrators, and policymakers as they seek out ways to lessen the burden of NAS. 

Our study has limitations as well, most of which are inherent to large administrative databases. As mentioned above the NIS does not allow to differentiate NICU admissions. Moreover, clinical data, as well as volume data, are not available which may act as possible confounders in the models. We did not exclude babies that may have required morphine for procedural sedation because under ICD-10-CM iatrogenic NAS is reported under separate codes.

## Conclusions

In newborns with neonatal abstinence syndrome, we found that each hospitalization day was associated with an increase of $1,685 in-hospital costs. Multiple co-morbidities and hospitalization at urban teaching hospitals were associated with increased length of stay and hospital cost. These have implications for improved care for NAS.
